# Physicochemical Properties and Hypolipidemic Activity of Dietary Fiber from Rice Bran Meal Obtained by Three Oil-Production Methods

**DOI:** 10.3390/foods12193695

**Published:** 2023-10-08

**Authors:** Renwei Zhu, Sha Tan, Yayi Wang, Linwei Zhang, Liang Huang

**Affiliations:** 1 School of Materials and Chemical Engineering, Tongren University, Tongren 554300, China; zhurenwei@126.com (R.Z.); tansha825325@126.com (S.T.); 2 School of Food Science and Engineering, Central South University of Forestry and Technology, Changsha 410004, China; yywang97@126.com (Y.W.); 20211100410@csuft.edu.cn (L.Z.); 3 Hunan Key Laboratory of Processed Food for Special Medical Purpose, Changsha 410004, China

**Keywords:** dietary fiber, rice bran meal, physicochemical properties, hypolipidemic activity

## Abstract

This study investigated the effects of three oil production methods on the physicochemical properties of dietary fiber from rice bran flour, and the hypolipidemic effects of the dietary fibers were investigated in vitro and in vivo. The particle size results showed that the organic-solvent-impregnated rice bran meal dietary fiber (N-RBDF) had the smallest average particle size and the aqueous enzymatic rice bran meal dietary fiber (E-RBDF) had the narrowest particle size distribution. Scanning electron microscopy (SEM) results demonstrated that all three kinds of rice bran meal dietary fibers (RBDFs) were irregularly flaky. Fourier transform infrared spectroscopy (FT-IR) results revealed that the three RBDFs had similar reactive groups, and X-ray diffraction (XRD) results indicated that all three RBDFs were cellulose type I crystals. The results of thermogravimetric analysis showed that the lignin content of N-RBDF was significantly lower than that of the other two. Among the three kinds of RBDFs, E-RBDF had higher water retention capacity, swelling capacity, oil holding capacity, and adsorption capacity for cholesterol and sodium bile salts. The results of experimental studies in hyperlipidemic rats showed that all three kinds of RBDFs significantly reduced triglycerides (TG), total cholesterol (TC), and low-density lipoprotein cholesterol (LDL-C) and elevated high-density lipoprotein cholesterol (HDL-C) in the serum of hyperlipidemic rats; they also significantly lowered malondialdehyde (MDA) and elevated total superoxide dismutase (T-SOD) and glutathione peroxidase (GSH-Px) activities in the livers of rats. In addition, all three kinds of RBDFs decreased aminotransferase (ALT) and aminotransferase (AST) activity in serum and also improved liver steatosis and reduced atherosclerosis index (AI) in rats with hyperlipidemia. Our study provides a reference for the development and utilization of rice bran meal and the application of rice bran meal dietary fiber in food processing.

## 1. Introduction

Hyperlipidemia is characterized by elevated serum total cholesterol (TC), low-density lipoprotein cholesterol (LDL-C), triglycerides (TG), or reduced high-density lipoprotein cholesterol (HDL-C), resulting in elevated serum lipoprotein level, hence the term hyperlipoproteinemia [1]. Hyperlipidemia leads to atherosclerosis, which in turn is one of the direct causes of cardiovascular diseases and ultimately leads to death due to blood clots and myocardial infarction [2]. As people’s living standards have improved, the consumption of refined food has increased, leading to the increase of “civil diseases” such as obesity, hyperlipidemia, hyperglycemia, cardiovascular diseases, cancer, and type 2 diabetes [3]. In clinical practice, lipid-lowering drugs such as Western statins and fibrates are the most common and effective drugs. However, they are associated with high cost and side effects [4]. Therefore, the search for natural lipid-lowering drugs has become a hot topic in recent years.

Rice bran meal (RBM) is the by-product of rice bran oil extraction, also known as defatted rice bran, which is stable and easy to store and process. In addition to retaining the nutritional values of rice bran, it is rich in protein, starch, and phytates, with dietary fiber content as high as 30 to 50% [5,6]. Dietary fiber is the sum of non-starch polysaccharides that cannot be digested and absorbed in the small intestine but can be fully or partially fermented during digestion in the large intestine [7]. According to its solubility, it can be divided into two major groups: soluble dietary fiber (SDF) and insoluble dietary fiber (IDF). SDF includes pectin, gum, and some other plant-related mucopolysaccharides [8]. IDF includes cellulose, hemicellulose, and lignin and exhibits high water-holding, swelling, and oil-holding properties as well as adsorption capacity, which suggests that IDF has the ability to increase stool volume, shorten the gastrointestinal transit cycle, and prevent colon cancer, obesity, type 2 diabetes, and cardiovascular diseases [9]. IDF contains free carboxyl and hydroxyl groups, and due to its high cation exchange capacity, IDF also has detoxifying effects [10]. Studies have shown that physical [11,12], chemical [13,14], and biological [15] methods can alter the structure of rice bran meal dietary fiber (RBDF), thus affecting its physicochemical and functional properties.

Among the extraction processes of vegetable oil, the most commonly used on an industrial scale are the pressing method and the leaching method, while aqueous enzymatic extraction is an emerging method. Different vegetable oil extraction processes have different effects on the quality of edible oils. Shi et al. [16] found that properties of the hot-pressed Torreya grandis kernel oils were superior to those of cold-pressed and solvent-extracted Torreya grandis kernel oils. Li et al. [17] studied the effects of different processing methods on the quality of rapeseed oil and animal lipid metabolism and found that the polyphenol, α-tocopherol, and β-carotene contents and antioxidant properties of canola oil extracted by the aqueous enzymatic method were better than those of cold-pressed, hot-pressed, and hexane-infused canola oil. Jiang et al. [18] investigated the effects of cold pressing, hot pressing, and enzyme-assisted aqueous extraction on the composition and nutrition of peanut oil and found that different process technologies caused differentiation of trace active ingredients. Wongwaiwech et al. [19] compared the bioactive substances in defatted rice bran derived from solvent-extracted and cold-pressed oil extraction and found that the content of γ-aminobutyric acid in the solvent-extracted defatted rice bran was higher than that of the cold-pressed residue. However, the effect of different oil extraction processes on the dietary fiber in rice bran meal has not been reported.

In this study, dietary fiber from rice bran meal obtained by different methods was prepared using a complex enzyme method. Then, the different RBDFs’ physicochemical and structural properties were compared. Last but not least, their potential hypolipidemic effect was evaluated in hyperlipidemic rats induced by a high-fat diet. This study provides a theoretical basis for the development and utilization of rice bran meal.

## 2. Materials and Methods

### 2.1. Materials

Rice bran was provided by Changsha Tianhao Grain Oil Co. (Changsha, China). Hydraulic-pressed rice bran meal (H-RBM), N-hexane-leached rice bran meal (N-RBM), and aqueous enzyme-method rice bran meal (E-RBM) were obtained after hydraulic pressing, hexane extraction, and aqueous enzyme production of rice bran oil in the laboratory. Heat-stable α-amylase (activity: 40,000 U/g), alkaline protease (activity: 200 U/mg), and glycosylase (activity: 100,000 U/g) were ordered from Shanghai Yuanye Biotechnology Co. (Shanghai, China). Sodium glycinate, sodium taurocholate, pepsin, and trypsin were obtained from Shanghai Maclean Biochemical Technology Co. (Shanghai, China). All other chemicals and solvents used were analytical grade.

### 2.2. Preparation of Samples

Rice bran meal dietary fiber (RBDF) was prepared according to Jia et al.’s [20] described method with a slight change. Briefly, 100 g of each of H-RBM, N-RBM and E-RBM were suspended in 1000 mL of distilled water. The suspension was hydrolyzed by 2.0 g heat-stable α-amylase for 60 min at 95 °C with an oscillation speed of 150 r/min; then, the enzyme was inactivated by cooling to 60 °C. After that, the pH of the suspension was adjusted to 8.0 by 1 mol/L NaOH solution. Proteins were removed by treatment with 2 g alkaline protease in a 60 °C water bath for 60 min. Lastly, the pH of the suspension was adjusted to 4.5 using 1 mol/L HCL solution, two gram of glycosylase was added, and the enzyme was hydrolyzed at 60 °C for 60 min with an oscillation speed of 150 r/min. Then, 4 times the volume of 95% ethanol precipitation was added. The mixture was stored overnight, and the residue obtained by filtration was dried and crushed to obtain hydraulic-pressed rice bran meal dietary fiber (H-RBDF), n-hexane-leached rice bran meal dietary fiber (N-RBDF), and aqueous enzyme-method rice bran meal dietary fiber (E-RBDF).

### 2.3. Structure Characterization

#### 2.3.1. Particle Size Distribution

The particle size distribution of RBDF samples was determined according to Bender et al.’s [21] method, using a Laser micron particle sizer (LS 13 320, Beckman Coulter Co., Brea, CA, USA).

#### 2.3.2. Scanning Electron Microscopy (SEM)

The micromorphological characteristics of the surfaces of H-RBDF, N-RBDF, and E-RBDF were observed via SEM (FEG-250, FEI Co., Hillsboro, OR, USA). The samples were scanned point by point with the focused electron beam under accelerating voltage of 20 kV and working distance to 11.4 mm.

#### 2.3.3. Fourier Transform Infrared (FT-IR) Spectroscopy

The functional groups and glycosidic bond types of H-RBDF, N-RBDF, and E-RBDF were detected using FTIR spectroscopy (IRTracer-100, Shimadzu, Japan). Samples of 2.0 mg of the dried H-RBDF, N-RBDF, and E-RBDF were weighed out, respectively. Then, each sample was put into a mill, and dried potassium bromide (KBr) powder was added 1:100 and ground well. Lastly, the sample was pressed into thin slices with a press, and scanned in the frequency region of 4000–400 cm^−1^ with blank KBr slices as the background.

#### 2.3.4. X-ray Diffraction Analysis (XRD)

According to the method by Hua et al. [22], with a slight change, the crystal structures of H-RBDG, N-RBDF, and E-RBDF were measured via X-ray diffractometer (D8 ADVANCE, Bruker Corporation, Billericay, MA, Germany) under diffraction conditions: Cu target, scanning area (2θ) 5°–60°, scanning speed 20°/min.

#### 2.3.5. Thermogravimetric Analysis (TGA)

The thermogravimetric (TG) and derivative thermogravimetric (DTG) analyses of H-RBDF, N-RBDF, and E-RBDF were determined using a TGA spectrometer (TGA/DSC 1/1100SF, METTLER TOLEDO, Zurich, Switzerland) with reference to the method by Zhang et al. [23] with slight modifications. A five milligram sample was weighed and loaded into an alumina crucible, and the temperature was controlled to increase from 30 °C to 600 °C at a heating rate of 10 °C/min. N_2_ was used as the protective gas.

### 2.4. Physicochemical Properties

#### 2.4.1. Water Holding Capacity (WHC)

The WHC of H-RBDF, N-RBDF, and E-RBDF was determined according to the method by Zhang et al. [24]. A one-gram sample was weighed into 80 mL of distilled water, shaken for 2 h at room temperature (25 °C), and centrifuged at 4000 r/min for 10 min. Then, the supernatant was discarded, the sample was weighed, and the WHC calculated according to formula:

(1) $$\mathrm{WHC}\;\left(g/g\right)=\frac{m_{2}-m_{1}-m_{0}}{m_{1}}$$

where WHC is the water holding capacity, g/g; *m*_0_ is the mass of the centrifuge tube, g; *m*_1_ is the mass of the sample before centrifugation, g; *m*_2_ is the sum of the mass of the sample and the centrifuge tube after centrifugation, g.

#### 2.4.2. Oil Holding Capacity (OHC)

The OHC of H-RBDF, N-RBDF, and E-RBDF was determined according to the method by Zhang et al. [24]. For this purpose, 1 g of sample and 40 mL of sesame oil were mixed in a centrifuge tube and shaken in a 25 °C water bath for 2 h, then the samples were centrifuged at 4000 r/min for 10 min. The upper layer of clear oil was discarded and weighed. The OHC was calculated according to formula:

(2) $$\mathrm{OHC}\;\left(g/g\right)=\frac{m_{2}-m_{1}-m_{0}}{m_{1}}$$

where OHC is the oil holding capacity, g/g; *m*_0_ is the mass of the centrifuge tube, g; *m*_1_ is the weight of the sample before centrifugation, g; *m*_2_ is the sum of the mass of the sample and the centrifuge tube after centrifugation, g.

#### 2.4.3. Swelling Capacity (SC)

A one-gram sample was weighed separately in a 10 mL graduated test tube, the volume of the sample was recorded, and 5.0 mL distilled water was added. The sample was stirred evenly and left to stand in a water bath at 25 °C for 24 h. The volume of dietary fiber after dissolution was recorded. SC was calculated according to formula:

(3) $$\mathrm{SC}\;\left(\mathrm{mL}/g\right)=\frac{V_{1}-V_{0}}{m}$$

where SC is the swelling force; m is the sample mass, g; *V*_0_ is the volume of the sample before swelling, mL; *V*_1_ is the volume of the sample after swelling, mL.

#### 2.4.4. Cholesterol Adsorption Capacity (CAC)

Referring to the method by Benitez et al. [25], slightly modified, the yolk from fresh eggs was diluted 1:10 (*v*/*v*) with deionized water. One gram of the sample was weighed in a conical flask and added to 25 g of the egg solution, the Ph was adjusted to 2.0 (simulated stomach environment) and 7.0 (simulated small intestine environment), respectively, and the samples were shaken at 150 r/min for 2 h at 37 °C. Then, the absorbance value was determined by the orthophthalaldehyde method. The cholesterol mass concentration c_1_ in the supernatant at 550 nm was measured, and the cholesterol mass concentration c_2_ in the egg yolk emulsion before adsorption was also measured. The CAC calculation formula is as follows:

(4) $$\mathrm{CAC}\left(\mathrm{mg}/g\right)=\frac{c_{2}-c_{1}}{M}\times V$$

where CAC is the cholesterol adsorption capacity, mg/g; c_2_ is the mass concentration of cholesterol before adsorption (mg/mL); c_1_ is the mass concentration of cholesterol after adsorption (mg/mL); M is the mass of sample (g); V is volume (mL).

#### 2.4.5. Sodium Cholate Binding Capacity (SCBC)

Referring to the method by Daou et al. [26], slightly modified, half a gram of sample was weighed and placed in a conical flask, then 1 mL of 0.01 mol/L HCl solution and 3 mL of 10 mg/mL pepsin solution were added, shaken, and digested in a water bath at 37 °C for 1 h. Subsequently, the pH was adjusted to 6.3 and 4.0 mL of 10 mg/mL trypsin solution was added, shaken, and digested at 37 °C for 1 h. Then, 4.0 mL of bile acid salt solution (0.5 mmol/L sodium taurocholate solution, 0.5 mmol/L sodium glycocholate solution, both prepared in PBS buffer at pH 6.3) was added, and the mixture was shaken in a water bath at 37 °C for 1 h and centrifuged at 4500 r/min for 20 min. Next, 2.4mL of supernatant was extracted, then 7.6 mL of 60% H_2_SO_4_ was added, the mixture was shaken well in a water bath at 70 °C for 20 min, and then in an ice bath for 5 min, and the absorbance was determined at 387 nm. The SCBC was then calculated as follows:

(5) $$\mathrm{sodium}\;\mathrm{glycocholate}\;\mathrm{binding}\;\mathrm{rate}\;\left(\%\right)=\frac{c_{3}-c_{4}}{c_{3}}$$

(6) $$\mathrm{sodium}\;\mathrm{taurocholate}\;\mathrm{binding}\;\mathrm{rate}\;\left(\%\right)=\frac{c_{5}-c_{6}}{c_{5}}$$

where *c*_3_ and *c*_4_ are the amounts of sodium glycocholate before and after adsorption (mmol); *c*_5_ and *c*_6_ are the amounts of sodium taurocholate before and after adsorption (mmol).

### 2.5. Animal Experiment Design

Forty-two specific-pathogen-free Sprague–Dawley rats weighing 180 ± 20 g were selected. Animals were provided by Hunan Silaikejingda Experimental Animal Co., Ltd. (Changsha, China). The animals were acclimatized and fed for one week at room temperature of 20–26 °C and humidity of 40–70%, with access to food and water freely. Then, they were randomly divided into 2 groups. In one group, 7 rats were given basic feed as a negative control, and the remaining 35 were given high-fat feed as the high-fat group. Serum total cholesterol (TC) and triglyceride (TG) of rats in the high-fat group were significantly higher compared with the NC group. The animal protocol was approved by Hunan Silaikejingda Experimental Animal Co., Ltd. Experimental Animal Welfare Ethics Committee (approval number: IACUC-SJA2022088) under the guidelines of the Chinese Academy of Sciences.

The 35 rats that were successfully modeled were randomized again and divided into 5 groups of 7 rats each; namely, the hyperlipidemia model (Model Control, MC) group, positive drug control (Positive Control, PC) group, H-RBDF group, N-RBDF group, and E-RBDF group. The negative control (NC) group was fed with basic feed, and the other groups were all fed high-fat feed. The amount of gavage according to Liu et al. [27] and details of feeding and dosing are shown in Table 1. All 6 groups of rats continued to be fed for 28 days with once-daily gavage. At the end of 4 weeks, all rats were fasted with food and water for 12 h. All rats were dissected after anesthesia with isoflurane, and the blood was quickly taken from the abdominal aorta. The livers were dissected, washed, dried, weighed, and photographed. One piece of liver was fixed in 4% paraformaldehyde and used for histopathological sections of the liver, and the rest were stored in a refrigerator at −80 °C for further analysis and detection.

### 2.6. Serum Lipid Level

The blood was centrifuged at 4000 r/min for 10 min, and the upper serum was used to determine TC, TG, HDL-C, and LDL-C with a fully automated biochemical analyzer (BS-430, Shenzhen Merry Biomedical Electronics Co., Shenzhen, China).

### 2.7. Determination of AST and ALT Activity in Serum

The rat serum was taken after centrifugation, and AST and ALT activities were determined according to the IFCC kit method using a fully automated biochemical analyzer (BS-430, Shenzhen Merry Biomedical Electronics Co., China).

### 2.8. Determination of GSH-Px, SOD Activity and MDA in Liver

Half a gram of liver tissue was put into a tissue grinder, and saline was added at 4 °C in the ratio of 1:9 (g/mL) by weight of liver. The liver tissue was ground into 10% homogenate in an ice–water bath and centrifuged at 3000 r/min for 10 min. The supernatant was extracted, and the GSH-Px, SOD, and MDA of the liver were determined using an enzyme marker (SpectraMax i3X, Molecular Devices, Silicon Valley, CA, USA) according to the kit instructions.

### 2.9. Pathological Observation of Liver

The rat livers were soaked in 4% paraformaldehyde fixative for 48 h, which was then replaced with new paraformaldehyde fixative for 24 h. The livers were then dehydrated with gradient ethanol, paraffin-embedded, dehydrated by hematoxylin and eosin section staining, sealed by neutral gum, and photographed under a panoramic slice scanner (PANNORAMIC DESK/MIDI/250/1000, 3DHISTECH, Budapest, Hungary).

### 2.10. Statistical Analyses

The results are shown as the mean ± standard deviation. The data were analyzed using Origin 2018 and SPSS 17.0 software. *p* values less than 0.05 were considered to be statistically significant.

## 3. Results and Discussion

### 3.1. Structure Analysis

#### 3.1.1. Particle Size Analysis

Both particle size and distribution can affect the structure and properties of dietary fibers. Therefore, many efforts have been made by researchers to modify dietary fibers, including the reduction of their particle size. Reducing the particle size of dietary fibers may lead to some changes in their structure, porosity, surface area, and functional properties [28]. The particle size and particle size distribution of H-RBDF, N-RBDF, and E-RBDF are shown in Table 2, respectively. The median particle size of N-RBDF was 18.10 μm, which was significantly smaller than that of E-RBDF (26.15 μm) and H-RBDF (39.82 μm); the specific surface area of N-RBDF (5613.72 m^2^/g) was also significantly larger than that of E-RBDF (3082.04 m^2^/g) and H-RBDF (1914.75 m^2^/g). The results indicate that the average particle size of N-RBDF is the smallest, E-RBDF is the second, and H-RBDF is the largest. However, the span of E-RBDF was 2.34, which was smaller than 2.61 for N-RBDF and 3.03 for H-RBDF, which indicates that the particle size distribution of E-RBDF was more uniform. A similar trend was observed in rice bran IDF [29].

#### 3.1.2. SEM Analysis

**Figure 1:**
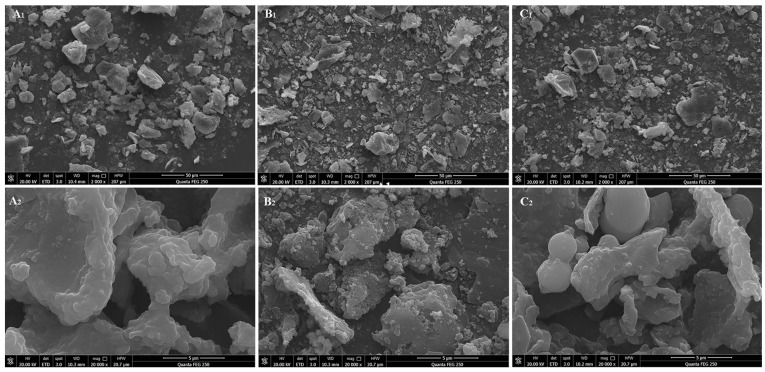
SEM of H-RBDF (**A_1_**, 2000×; **A_2_**, 20,000×), N-RBDF (**B_1_**, 2000×; **B_2_**, 20,000×), and E-RBDF (**C_1_**, 2000×; **C_2_**, 20,000×). H-RBDF: hydraulic-pressed rice bran meal dietary fiber; N-RBDF: n-hexane leached rice bran meal dietary fiber; E-RBDF: enzyme-method rice bran meal dietary fiber.

The SEM images of H-RBDF, N-RBDF, and E-RBDF are illustrated in Figure 1. It can be seen from Figure 1A_1_,B_1_,C_1_ that the N-RBDF particles were smaller; n-hexane treatment can destroy the structure of rice bran to make it more fragmented, dried, and cracked [30]. It can be seen from Figure 1A_2_,B_2_,C_2_ that H-RBDF, N-RBDF, and E-RBDF were all irregularly lamellar with protein particles on the surface. While N-RBDF had the most protein particles attached to the surface, a broken lamellar structure, and a larger specific surface area, and E-RBDF had more folds and pore-like structures on the surface, H-RBDF had a smooth surface with more particles attached. The pleated and pore-like structure of the dietary fiber not only increased its water and oil holding power, but also raised its adsorption capacity [31].

#### 3.1.3. FT-IR Analysis

**Figure 2:**
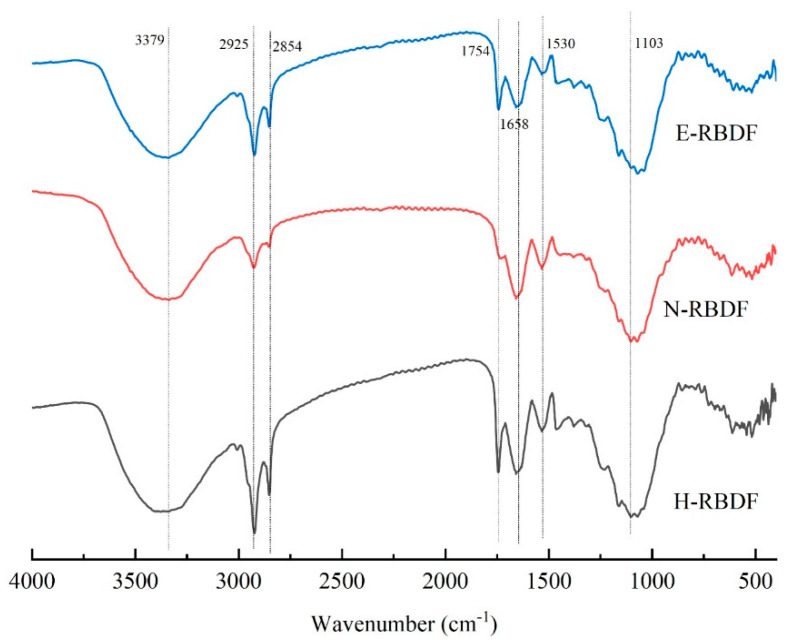
Fourier infrared spectrum of H-RBDF, N-RBDF, and E-RBDF. H-RBDF: hydraulic-pressed rice bran meal dietary fiber; N-RBDF: n-hexane-leached rice bran meal dietary fiber; E-RBDF: enzyme-method rice bran meal dietary fiber.

Figure 2 shows the FTIR chart of H-RBDF, N-RBDF, and E-RBDF; the absorption peaks near the wave number 3379 cm^−1^ in the IR spectrogram are intramolecular or intermolecular hydrogen bond stretching vibrations of the -OH group within the crystalline region of cellulose [32]. The two absorption peaks at 2925 cm^−1^ and 2854 cm^−1^ are due to the methyl or methylene groups, resulting in the typical C-H stretching band [33]. The peak near 1754 cm^−1^ is caused by the characteristic peak of the stretching vibration of the ester C=O [34]. The absorption peak at 1658 cm^−1^ may be a characteristic peak caused by the (-NHZ) variable angle vibration, or it may be caused by the carboxyl stretching vibration of the hydrogen bonds formed between the cellulose chains [35]. The stretching vibration absorption peak of carboxyl C=O appears near 1530 cm^−1^, which could also be an absorption peak caused by asymmetric stretching vibrations, and most of the absorption peaks responding to these positions are characteristic absorption peaks of sugars [36]. The peak near 1103 cm^−1^ is probably caused by the C-O stretching vibration of C-O-C between hemicellulose and cellulose [37]. In summary, the position and number of characteristic absorption peaks of H-RBDF, N-RBDF, and E-RBDF did not change significantly after the modification treatment, but the intensity of the absorption peaks varied. Especially, the intensity of N-RBDF at 2925 cm^−1^ and 1754 cm^−1^ was significantly weakened, probably due to the destruction of ester, ether, and intermolecular hydrogen bonding groups of cellulose, lignin, and hemicellulose during the organic solvent extraction [38].

#### 3.1.4. XRD Analysis

**Figure 3:**
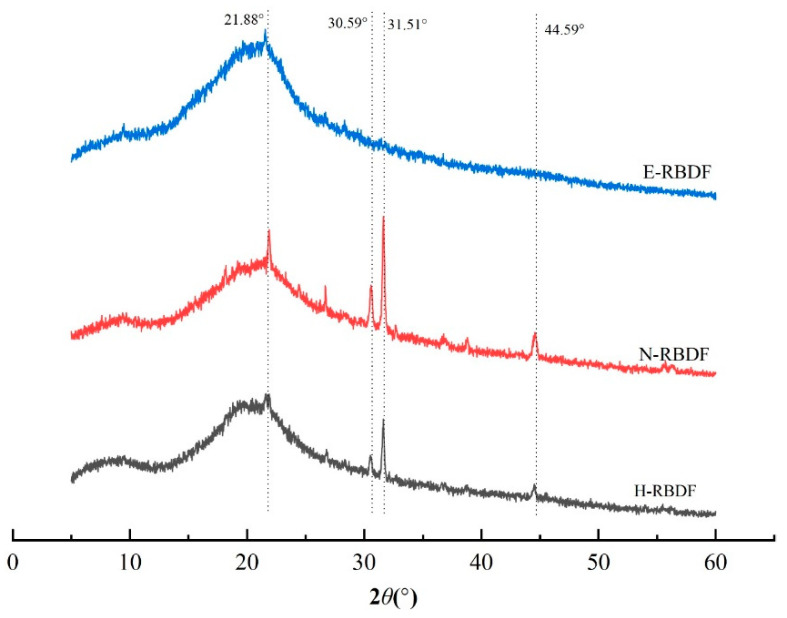
XRD patterns of H-RBDF, N-RBDF, and E-RBDF. H-RBDF: hydraulic-pressed rice bran meal dietary fiber; N-RBDF: n-hexane-leached rice bran meal dietary fiber, E-RBDF: enzyme-method rice bran meal dietary fiber.

It can be seen from Figure 3 that H-RBDF, N-RBDF, and E-RBDF all have a strong diffraction peak near 2θ at 21.88°, indicating that they are typical cellulose I crystals [39]. Both H-RBDF and N-RBDF have strong diffraction peaks near 31.54° and weaker diffraction peaks at 30.59° and 44.59° [40], which might be a result of organic solvents and hydraulic pressure causing changes in the crystal structures of H-RBDF and N-RBDF. The degrees of crystallinity of H-RBDF, N-RBDF, and E-RBDF were calculated using MDI Jade v 6.0 software as 31.98%, 20.27%, and 29.80%, respectively, and the decrease in crystallinity might have been caused by the organic solvents and enzymes that disrupt the crystalline regions of rice bran meal dietary fibers more significantly than hydraulic pressure [41].

#### 3.1.5. Thermal Analysis

**Figure 4:**
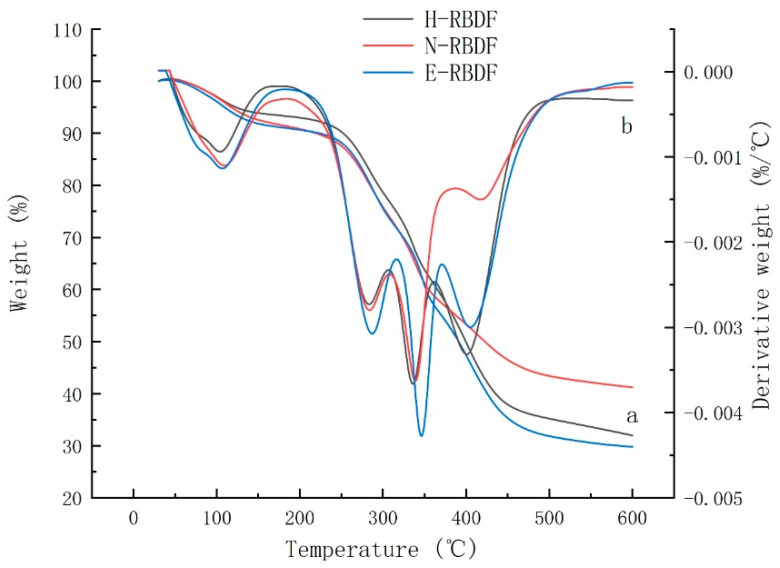
Thermogravimetric (a) and differential thermogravimetric (b) curves of H-RBDF, N-RBDF, and E-RBDF. H-RBDF: hydraulic-pressed rice bran meal dietary fiber; N-RBDF n-hexane-leached rice bran meal dietary fiber; E-RBDF: enzyme-method rice bran meal dietary fiber.

Thermogravimetric (TG) and differential thermogravimetric (DTG) analyses were used to evaluate the changes in thermal stability and structural properties of H-RBDF, N-RBDF, and E-RBDF. Lignin, cellulose, and hemicellulose in dietary fibers have different thermal decomposition temperatures due to their different chemical structures [42]. Curve a in Figure 4 shows the thermogravimetric curves of H-RBDF, N-RBDF, and ERBDF; the solid residues of H-RBDF, N-RBDF, and E-RBDF were 31.98%, 41.21% and 29.79%, respectively. The results indicate that the chemical method improved the thermal stability of RBDF, which was consistent with the results of the study by Zhang et al. [43]. The DTG curves of H-RBDF, N-RBDF, and E-RBDF (Figure 4b) have four peaks. The first peak at around 100 °C might be due to evaporation of water; the second peak at 289 °C might be due to degradation of hemicellulose. Moriana et al. [44] showed that hemicellulose usually degrades rapidly between 210 and 350 °C. The third peak, located at 336 °C, was formed by cellulose degradation. Yang et al. [45] found that cellulose degradation starts at 315 °C and continues until 400 °C and the maximum loss rate of cellulose peaks at 355 °C, which was also reported by Kazachenko et al. [46]. The fourth peak, located at 430 °C, was caused by lignin degradation, and both hemicellulose and cellulose were degraded below 400 °C. Zhang et al. [47] reported that the maximum rate of lignin loss occurred between 399–450 °C. The fourth peak of N-RBDF was significantly weaker than that of H-RBDF or E-RBDF, and this result indicates that the lignin content of H-RBDF and E-RBDF was higher than that of N-RBDF, which might be caused by the destruction of the structure of lignin during the organic solvent extraction.

### 3.2. Functional Properties Analysis

#### 3.2.1. WHC, OHC, and SC Analysis

The WHC and SC of dietary fiber are important properties in food processing. Higher WHC can bring better taste and processing properties to foods, stronger SC can promote gastrointestinal motility and defecation and increase the volume of feces, thus having the function of preventing constipation, and the particle size, specific surface area, and hydrophilic group content of dietary fiber particles can affect their WHC and SC [48,49]. The porous microstructure and larger specific surface area of dietary fibers can increase the contact between fibers and oil and improve the oil-holding power. However, too small particle size will instead expose more hydrophilic groups thereby reducing the OHC [50], and higher OHC is beneficial for dietary fibers to absorb excess oil in the human body, which can prevent obesity. The WHC, OHC, and SC of H-RBDF, N-RBDF, and E-RBDF are shown in Table 3. The WHC, OHC, and SC of E-RBDF reached 2.39 g/g, 1.61 g/g, and 1.59 mL/g, which were 1.56 times, 1.27 times, and 1.33 times those of H-RBDF and 1.29 times, 1.1 times, and 1.37 times those of N-RBDF, respectively. 

#### 3.2.2. CAC Analysis

**Figure 5:**
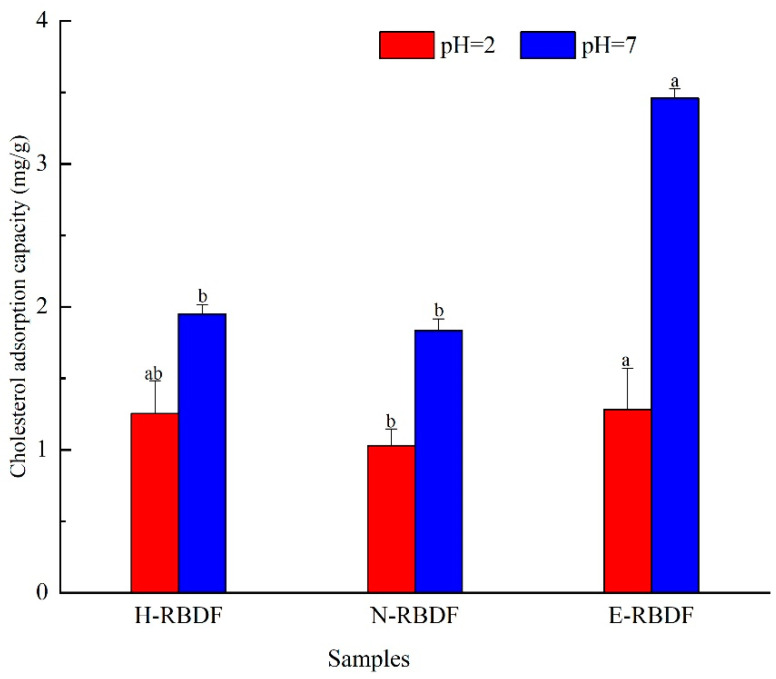
Cholesterol adsorption capacity of H-RBDF, N-RBDF, and E-RBDF. The values represent mean ± SD (n = 3) and different letters show significant difference at the *p* < 0.05 level. H-RBDF: hydraulic-pressed rice bran meal dietary fiber; N-RBDF: n-hexane-leached rice bran meal dietary fiber; E-RBDF: enzyme-method rice bran meal dietary fiber.

The cholesterol adsorption capacities of H-RBDF, N-RBDF, and E-RBDF are shown in Figure 5. It can be seen that the cholesterol adsorption capacity of rice bran meal dietary fiber was stronger at pH = 7 than at pH = 2 (*p* < 0.05), which indicates that rice bran meal dietary fiber is mainly used for cholesterol adsorption in the intestine, which is consistent with the findings of Nsor-Atindana et al. [51]. The cholesterol adsorption capacity of E-RBDF was 1.02 times that of H-RBDF and 1.24 times that of N-RBDF at pH = 2, 1.77 times that of H-RBDF, and 1.89 times that of N-RBDF at pH = 7, respectively. These results are consistent with the conclusion that E-RBDF had more folds and pore-like structures on the surface, which made the dietary fiber more adsorbent to cholesterol.

#### 3.2.3. SCBC Analysis

**Figure 6:**
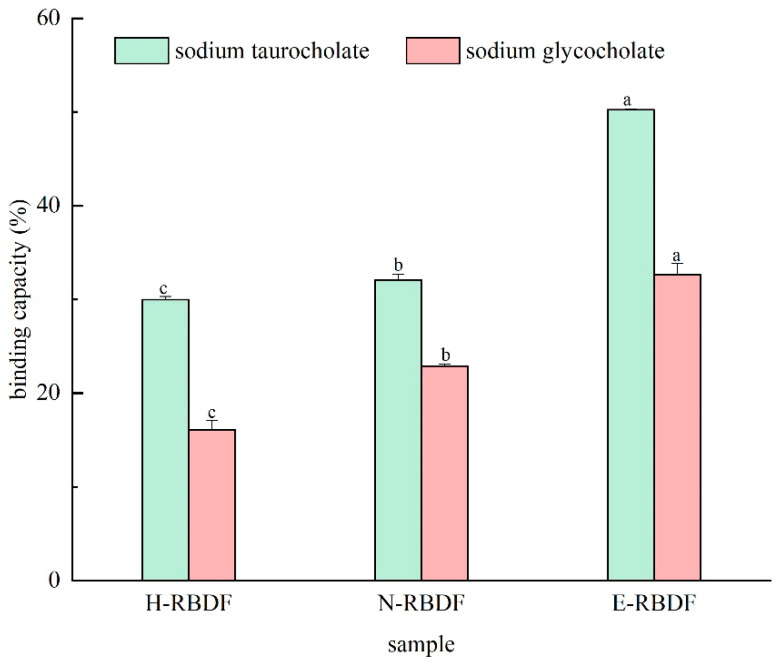
In vitro adsorption capacities to sodium cholate binding capacity of H-RBDF, N-RBDF, and E-RBDF. The values represent mean ± SD (n = 3). Different letters are significantly different at the level of *p* < 0.05. H-RBDF: hydraulic-pressed rice bran meal dietary fiber; N-RBDF: n-hexane-leached rice bran meal dietary fiber; E-RBDF: enzyme-method rice bran meal dietary fiber.

Dietary fiber combined with bile acid sodium salt is an indirect way to lower cholesterol and blood lipids, because the increased egestion of bile acid sodium salt can facilitate the consumption of cholesterol metabolite bile acid, further accelerating cholesterol decomposition and lowering serum cholesterol levels [52]. The adsorption capacities of H-RBDF, N-RBDF, and E-RBDF for sodium glycocholate and sodium taurocholate are shown in Figure 6, with the binding rate of sodium glycocholate reaching (32.61 ± 1.21)% for E-RBDF, which is 2.03 times higher than that of H-RBDF and 1.43 times higher than that of N-RBDF. The sodium taurocholate binding rate of E-RBDF reached (50.24 ± 0.09)%, which is 1.68 times higher than that of H-RBDF and 1.57 times higher than that of N-RBDF.

### 3.3. Effect of Dietary Fiber on Blood Lipid Levels of Rats

As shown in Table 4, TC, TG, and LDL-C levels in the MC group were significantly higher than those in the NC group (*p* < 0.01), and HDL-C levels were lower than those in the NC group (*p* < 0.05). Compared with the MC group, TG was highly significantly lower in the H-RBDF, N-RBDF, and E-RBDF groups (*p* < 0.01), TC was significantly lower in the H-RBDF and E-RBDF groups (*p* < 0.05), TC was also lower in the N-RBDF group, LDL-C was significantly lower in the H-RBDF, N-RBDF, and E-RBDF groups (*p* < 0.05), and HDL-C levels were essentially unchanged. The above results indicate that H-RBDF, N-RBDF, and E-RBDF can improve the accumulation of lipids in the serum of hyperlipidemic rats and have some hypolipidemic activity. Especially, E-RBDF was more effective than H-RBDF and N-RBDF in reducing TG and TC levels in the serum of hyperlipidemic rats. Hu et al. [53] found that xylanase-modified corn husk fiber can significantly reduce TG, TC and LDL-C in serum of rats. The AI of hyperlipidemic rats in the MC group was extremely significantly higher than that in the NC group, indicating that feeding a high-fat diet led to elevated AI in rats. The AI of hyperlipidemic rats in the PC group, H-RBDF group, N-RBDF group, and E-RBDF group decreased to different degrees. In addition, the AI of rats in the H-RBDF and E-RBDF groups was significantly smaller than that in the MC group (*p* < 0.05), and the effect of E-RBDF intervention treatment was the best. Similarly, Dabour et al. [54] found that yogurt supplemented with wheat bran or dietary fiber significantly (*p* < 0.05) reduced AI in hypercholesterolemic rats.

### 3.4. Effect of Dietary Fiber on AST and ALT Activity in Serum

**Figure 7:**
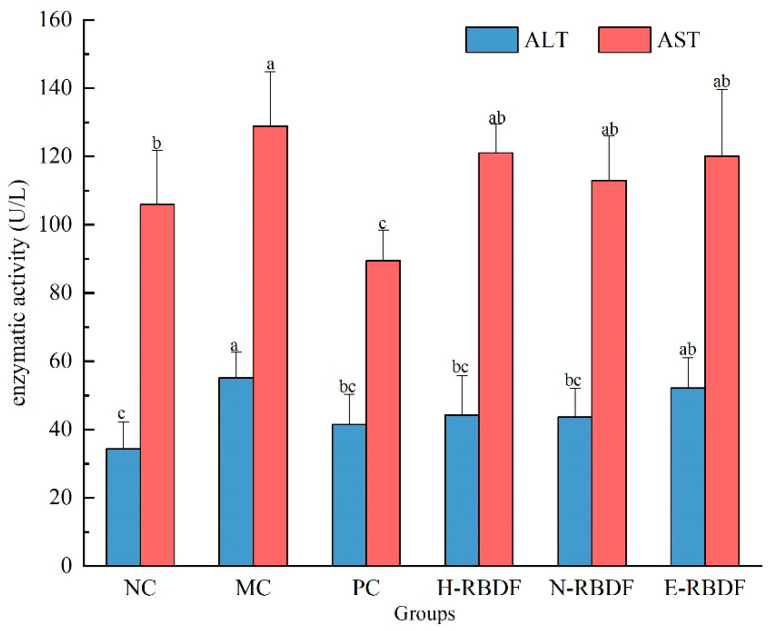
Effects of three types of rice bran meal dietary fiber on AST and ALT levels in rat serum. The values represent mean ± SD (n = 7), and different letters show significant difference at the *p* < 0.05 level. NC, MC, and PC are negative control group, model control group, and positive control group, respectively. H-RBDF, N-RBDF, and E-RBDF are the gavaged drug-intervention groups: hydraulic-pressed rice bran meal dietary fiber, n-hexane-leached rice bran meal dietary fiber, enzyme-method rice bran meal dietary fiber, respectively.

ALT and AST are dysfunctional enzymes found in serum and plasma, mainly in hepatocytes, and their activity is often used to determine liver injury or liver dysfunction [55]. When hepatocytes are damaged, ALT and AST are released into the blood, the enzyme activity increases, and this increase in ALT and AST activity is a sign of liver damage [56]. The activities of ALT and AST in the serum of rats are shown in Figure 7. ALT and AST were highly significantly elevated in the MC group compared with the NC group (*p* < 0.01), indicating that feeding a long-term high-fat diet leads to liver damage in rats. After drug, H-RBDF, N-RBDF, and E-RBDF interventions, the PC group, H-RBDF group, N-RBDF group, and E-RBDF group all showed reduced ALT and AST activities in the serum of hyperlipidemic rats.

### 3.5. Effect of Dietary Fiber on GSH-Px, SOD Activity, and MDA in the Liver

GSH-Px is an enzyme that catalyzes the breakdown of H_2_O_2_, scavenging lipid peroxides and blocking the chain reaction of lipid peroxidation, thus achieving the reduction of hepatocyte damage [57]. SOD can effectively scavenge oxygen free radicals in the body and maintain the balance of the body’s oxidative and antioxidant capacity, and SOD activity is one of the main indicators of the body’s antioxidant capacity [58]. MDA is a membrane lipid peroxidation product that indirectly reflects the damage of hepatocytes in fatty liver patients in a state of lipid peroxidation and oxidative stress [59]. It can be seen from Table 5 that the MC group had significantly reduced activity of GSH-Px and SOD in the livers of hyperlipidemic rats relative to the NC group (*p* < 0.01), and also significantly elevated the MDA content in the livers of hyperlipidemic rats (*p* < 0.01). Compared with the MC group, the GSH-Px activity in the livers of hyperlipidemic rats in the PC, H-RBDF, N-RBDF, and E-RBDF groups was highly significantly increased (*p* < 0.01), and the SOD activity was also raised. In addition, the MDA content in the livers of hyperlipidemic rats in the PC, H-RBDF, N-RBDF, and E-RBDF groups highly significantly decreased (*p* < 0.01). In conclusion, H-RBD, N-RBDF, and E-RBDF enhanced the antioxidant activity in the livers of hyperlipidemic rats, thereby reducing liver damage in rats. Similarly, Liu et al. [60] found that dietary fiber from monkey head mushroom significantly counteracted oxidative stress by reducing lipid peroxidation product levels and promoting enzymatic and non-enzymatic antioxidant activities.

### 3.6. Histopathological Analysis of the Rat Liver

**Figure 8:**
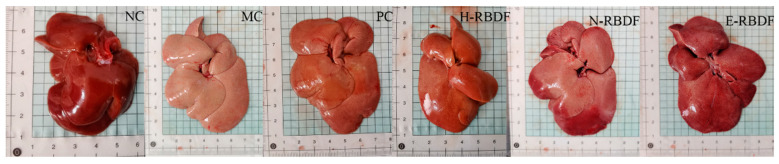
Effects of three types of rice bran meal dietary fiber on the appearance of the liver in hyperlipidemic rats. NC, MC, and PC are negative control group, model control group, and positive control group, respectively. H-RBDF, N-RBDF, and E-RBDF are the gavaged drug-intervention groups: hydraulic-pressed rice bran meal dietary fiber, n-hexane-leached rice bran meal dietary fiber, enzyme-method rice bran meal dietary fiber, respectively.

Figure 8 shows the appearance of the livers of hyperlipidemic rats. The livers of rats in the NC group were dark red in color with smooth and shiny surfaces and sharp edges, while the livers of rats in the MC group were significantly larger in size and creamy white in color, with blunt edges and fat particles visible to the naked eye on the surface. The livers of rats in the PC, H-RBDF, N-RBDF, and E-RBDF groups were significantly improved in color and also had improved fat particles on the surface compared with the MC group, but there was no significant decrease in volume.

**Figure 9:**
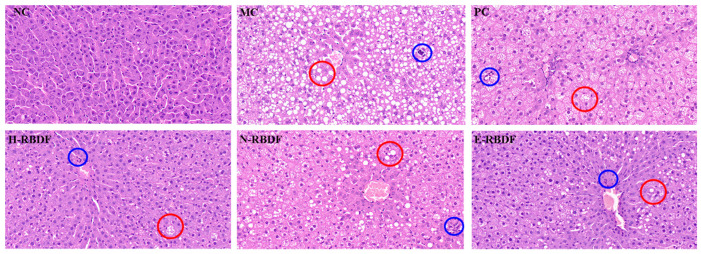
Effects of three types of rice bran meal dietary fiber on pathological changes in the livers of hyperlipidemic rats. NC, MC, and PC are negative control group, model control group, and positive control group, respectively. H-RBDF, N-RBDF, and E-RBDF are the gavaged drug-intervention groups: hydraulic-pressed rice bran meal dietary fiber, n-hexane-leached rice bran meal dietary fiber, enzyme-method rice bran meal dietary fiber, respectively.

As shown in Figure 9, the hepatocytes in the livers of the NC group were structurally intact, densely arranged, and unchanged, while the hepatocytes in the livers of the MC group were covered with fat particles of different sizes (red circle), with diffuse steatosis and disorganized cell arrangement, and small focal necrosis of the hepatocytes was seen, accompanied by a small amount of inflammatory cell infiltration (blue circle). This indicated that steatosis and histopathology in the rat liver were due to feeding a high-fat diet. Compared with the MC group, the above symptoms were improved in the livers of rats in the PC, H-RBDF, N-RBDF, and E-RBDF groups, but a small amount of white fat particles were still visible (red circle). In contrast, the fat particles in the livers of rats in the H-RBDF and E-RBDF groups were significantly less than those in the N-RBDF. The improvement of cellular arrangement in the livers of rats in the E-RBDF group was more obvious compared with those in the H-RBDF and N-RBDF groups, and these results were consistent with the results of the liver appearance maps for the rats in each group. The above results show that all three kinds of rice bran meal dietary fiber could improve the fibrosis phenomenon in the livers of rats with hyperlipidemia, and had a certain positive intervention effect on the degeneration of fat in the livers of rats, with a certain hypolipidemic function.

## 4. Conclusions

In this study, the effects of three oil production methods on the physicochemical properties and lipid-lowering function of rice bran meal dietary fiber were investigated. All three kinds of rice bran meal dietary fibers showed irregular flakes, exhibited polysaccharide infrared absorption spectral characteristics, and showed characteristic cellulose type I diffraction peaks. E-RBDF had higher water holding capacity, oil holding capacity, swelling capacity, cholesterol adsorption capacity, and sodium cholate adsorption capacity compared with H-RBDF and N-RBDF. It is possible that enzymes can better expose the functional groups of RBDFs and disrupt the lamellar structure of RBDFs to form uniformly sized particles. Animal experiments showed that all three kinds of rice bran meal dietary fiber could reduce TC, TG, LDL-C, AI, AST, and ALT activity and enhance HDL-C in serum of hyperlipidemic rats; they could also enhance GSH-Px and SOD activity in liver tissues of hyperlipidemic rats and at the same time reduce MDA content in liver tissues of hyperlipidemic rats, alleviate liver damage, and reduce steatosis. Among them, E-RBDF had the best therapeutic effect on hyperlipidemic rats, which was consistent with the result that E-RBDF had better physicochemical properties. This study can provide a reference for the selection of rice bran oil extraction methods and the development and utilization of rice bran meal. However, the lipid-lowering mechanism of RBDF needs to be further investigated.

## Figures and Tables

**Table 1:** Feeding and drug administration for each group of rats.

Group	Feed	Gavage Drugs
NC	Basic feed	Normal saline
MC	High-fat feed	Normal saline
PC	High-fat feed	2 mg/Kg BW/d simvastatin
H-RBDF	High-fat feed	3 g/Kg BW/d H-RBDF
N-RBDF	High-fat feed	3 g/Kg BW/d N-RBDF
E-RBDF	High-fat feed	3 g/Kg BW/d E-RBDF

Note: NC, MC, and PC are negative control group, model control group, and positive control group, respectively. H-RBDF, N-RBDF, and E-RBDF are the gavaged drug-intervention groups: hydraulic-pressed rice bran meal dietary fiber, n-hexane-leached rice bran meal dietary fiber, enzyme-method rice bran meal dietary fiber, respectively.

**Table 2:** Particle size distribution of H-RBDF, N-RBDF, and E-RBDF.

Sample	Specific Surface Area m^2^/g	D [3,2] (μm)	D10 (μm)	D50 (μm)	D90 (μm)	Span
H-RBDF	1914.75	31.34	15.94	39.82	136.68	3.03
N-RBDF	5613.72	10.68	4.68	18.10	51.95	2.61
E-RBDF	3082.04	19.47	9.35	26.15	70.72	2.34

Note: D [3,2] denotes the area-averaged diameter of the particles; D10, D50, and D90 denote the particle size corresponding to a sample with a cumulative particle size distribution of 10%, 50%, and 90%; Span is the span: $Span=\frac{D90-D10}{D50}$. H-RBDF: hydraulic-pressed rice bran meal dietary fiber; N-RBDF: n-hexane-leached rice bran meal dietary fiber; E-RBDF: enzyme-method rice bran meal dietary fiber.

**Table 3:** WHC, OHC, and SC of H-RBDF, N-RBDF, and E-RBDF.

Samples	WHC (g/g)	OHC (g/g)	SC (mL/g)
N-RBDF	1.85 ± 0.05 ^b^	1.46 ± 0.00 ^b^	1.19 ± 0.00 ^b^
E-RBDF	2.39 ± 0.01 ^a^	1.61 ± 0.01 ^a^	1.59 ± 0.01 ^a^
H-RBDF	1.53 ± 0.02 ^c^	1.26 ± 0.02 ^c^	1.20 ± 0.01 ^b^

Note: Data are presented as the mean values ± SD (*n* = 3). Different letters in the same column indicate significant differences *p* < 0.05. H-RBDF: hydraulic-pressed rice bran meal dietary fiber; N-RBDF: n-hexane-leached rice bran meal dietary fiber; E-RBDF: enzyme-method rice bran meal dietary fiber.

**Table 4:** Effects of three types of rice bran meal dietary fiber on TG, TC, HDL-C, LDL-C, and AI in the serum of hyperlipidemic rats.

Groups	TC (mmol/L)	TG (mmol/L)	HDL-C (mmol/L)	LDL-C (mmol/L)	AI
NC	0.71 ± 0.19	1.59 ± 0.19	1.02 ± 0.20	0.34 ± 0.09	0.58 ± 0.17
MC	1.97 ± 0.80 ^ΔΔ^	2.63 ± 0.67 ^ΔΔ^	0.78 ± 0.13 ^Δ^	0.83 ± 0.33 ^ΔΔ^	2.44 ± 0.95 ^ΔΔ^
PC	0.95 ± 0.37 **	2.38 ± 0.37	0.76 ± 0.15	0.81 ± 0.20 *	2.16 ± 0.44
H-RBDF	1.01 ± 0.31 **	2.15 ± 0.22 *	0.79 ± 0.07	0.70 ± 0.10 *	1.72 ± 0.31 *
N-RBDF	0.95 ± 0.26 **	2.23 ± 0.37	0.73 ± 0.15	0.69 ± 0.17 *	2.09 ± 0.40
E-RBDF	0.76 ± 0.18 **	1.97 ± 0.41 *	0.78 ± 0.16	0.74 ± 0.14 *	1.55 ± 0.46 *

Note: Data are presented as the mean values ± SD (n = 7); compared with NC group, ^Δ^ *p* < 0.05, ^ΔΔ^ *p* < 0.01; compared with MC group, * *p* < 0.05, ** *p* < 0.01. NC, MC, and PC are negative control group, model control group, and positive control group, respectively. H-RBDF, N-RBDF, and E-RBDF are the gavaged drug-intervention groups: hydraulic-pressed rice bran meal dietary fiber, n-hexane-leached rice bran meal dietary fiber, enzyme-method rice bran meal dietary fiber, respectively.

**Table 5:** Effects of three types of rice bran meal dietary fiber on GSH-Px, SOD, and MDA in rat liver.

Groups	GSH-Px (U/mgprot)	SOD (U/mgprot)	MDA (nmol/mgprot)
NC	102.7 ± 10.16	32.2 ± 5.17	26.9 ± 3.36
MC	50.0 ± 8.96 ^ΔΔ^	5.6 ± 1.50 ^ΔΔ^	151.5 ± 21.13 ^ΔΔ^
PC	76.4 ± 13.66 **	6.9 ± 1.74	56.1 ± 10.73 **
H-RBDF	72.2 ± 12.36 **	8.8 ± 2.83 *	78.7 ± 15.66 **
N-RBDF	67.7 ± 9.86 *	7.2 ± 1.50	82.6 ± 10.44 **
E-RBDF	86.9 ± 5.73 **	7.8 ± 1.52	63.7 ± 14.64 **

Note: Data are presented as the mean values ± SD (n = 7); compared with NC group, ^ΔΔ^ *p* < 0.01; compared with MC group, * *p* < 0.05, ** *p* < 0.01. NC, MC, and PC are negative control group, model control group, and positive control group, respectively. H-RBDF, N-RBDF, and E-RBDF are the gavaged drug-intervention groups: hydraulic-pressed rice bran meal dietary fiber, n-hexane-leached rice bran meal dietary fiber, enzyme-method rice bran meal dietary fiber, respectively.

## Data Availability

Data are contained within the article.

## Abbreviations

TDF	Total dietary fiber
SDF	Soluble dietary fiber
IDF	Insoluble dietary fiber
H-RBM	Hydraulic-pressed rice bran meal
N-RBM	N-hexane--leached rice bran meal
E-RBM	Enzyme-method rice bran meal
H-RBDF	Hydraulic-pressed rice bran meal dietary fiber
N-RBDF	N-hexane-leached rice bran meal dietary fiber
E-RBDF	Enzyme-method rice bran meal dietary fiber
NC	Negative control
MC	Model control
PC	Positive control
MDA	Malonic dialdehyde
SOD	Superoxide dismutase
GSH-Px	Glutathione peroxidase
AST	Aspartate aminotransferase
ALT	Alanine aminotransferase
TG	Triacylglycerol
TC	Total cholesterol
HDL-C	High-density lipoprotein cholesterol
LDL-C	Low-density lipoprotein cholesterol
AI	Atherogenic index
